# Metabolite profiling of tubers of an early- and a late-maturing potato line and their grafts

**DOI:** 10.1007/s11306-022-01950-3

**Published:** 2022-11-05

**Authors:** Vanda Villányi, Orsolya Kinga Gondor, Zsófia Bánfalvi

**Affiliations:** 1 Genetics and Biotechnology Institute, Hungarian University of Agriculture and Life Sciences, Szent-Györgyi A. u. 4, Gödöllő, 2100 Hungary; 2 Centre for Agricultural Research, Eötvös Loránd Research Network, Brunszvik u. 2, Martonvásár, 2462 Hungary

**Keywords:** *Solanum tuberosum*, GC–MS, Tuber, Grafting, Metabolic profiling

## Abstract

**Introduction:**

Earliness of tuberisation and the quality of potato tubers are important traits in potato breeding. The qualitative traits rely on the metabolite profile of tubers, which are storage organs and net importers of assimilates. Thus, the quality of tubers largely depends on the metabolites transported from leaves to developing tubers.

**Objectives:**

To test the influence of canopy on the quality of tubers by metabolite profiling of tubers of an early- and a late-maturing potato line and their grafts.

**Methods:**

Potatoes were grown under greenhouse conditions, grafted and the tubers harvested at the end of the scions’ vegetation period. Metabolite profiling of freshly harvested tubers was performed using gas chromatography coupled with mass spectrometry. Statistical analyses were applied to determine the significant differences between the different tubers.

**Results:**

99 metabolites were identified and an additional 181 peaks detected in chromatograms, out of which 186 were polar and 94 non-polar compounds. The concentrations of 113 metabolites were significantly different in the tubers from the early-maturing CE3130 and the late-maturing CE3027 line. Hetero-grafting resulted in considerable changes in the metabolite content of tubers. Especially, the effect of CE3027 on the metabolite composition of tubers formed on CE3130 rootstocks was readily apparent. Nevertheless, many compounds were present at similar levels in the tubers of hetero-grafted plants as was found in the tubers of their scion counterparts.

**Conclusion:**

Hetero-grafting resulted in many compounds at similar concentrations in rootstock tubers as in scion tubers suggesting that these are transported from the source leaves to tubers.

**Supplementary Information:**

The online version contains supplementary material available at 10.1007/s11306-022-01950-3.

## Introduction

Customers demand good taste, flavour, colour and nutrient quality from commercial potatoes. These qualitative traits rely on the metabolic profile of tubers, which typically contain several hundreds of different metabolites present at a wide concentration range (Beckmann et al., 2007). Gas chromatography coupled with mass spectrometry (GC–MS) was first applied by Roessner et al. (2000) for metabolite fingerprinting of potato tubers. As this technique proved reproducible, highly sensitive and capable of quantifying many metabolites, it has been applied for the characterisation of several varieties and breeding lines as well as to determining the influence of the environment on the quality traits of tubers. Dobson et al. (2008), for example, analysed 27 tetraploid potato varieties including native Chilean, old European and modern breeding varieties and found that the varieties differed in the content of a number of polar and non-polar compounds. The metabolomics approach has also been applied to monitoring metabolites during the tuber life cycle and upon storage (Sepherd et al., 2010; Uri et al., 2014). Metabolomics combined with ionomics detected 2656 compounds in potato tubers including bioactives, nutrients, lipids and metals, nevertheless, only 184 out of them were identified (Chaparro et al., 2018). Fukuda et al. (2019) demonstrated that GC–MS could be used even for metabolomics-based prediction of potato tuber sprouting during long-term storage. Metabolomics enabled identifying biomarkers of tuber black dot-causing *Colletotrichum coccodes* infection by detecting constitutive and induced resistance-related metabolites (Massana-Codina et al., 2020).

Potato tubers are formed from underground stems called stolons. In terms of maturity type, early-, middle- and late-maturing genotypes are distinguished. Maturity is an important breeding trait because early-maturing potato cultivars complete their life cycle before stress becomes a serious constraint and, therefore, are more profitable for growers. Several studies have identified QTLs for maturity on chromosome V (Bradshaw et al., 2008; Collins et al., 1999; Hackett et al., 2014; Li et al., 2018; Sliwka et al., 2007). Kloosterman et al. (2013) narrowed down the locus responsible for this trait to a 110-kb region on chromosome V and identified the *CYCLING DOF FACTOR* (*CDF*) family gene, *StCDF1*, as a key gene for the plant maturity phenotype based on the allelic differences of the gene between the very late-maturing CE3027 and the very early maturing CE3130 line, both derived from the same C × E diploid potato cross.

Early tuberisation, however, may affect the quality of tubers. To test this assumption, we used the same diploid potato lines, CE3027 and CE3130, in grafting experiments that were used earlier by Kloosterman et al. (2013) and analysed the metabolome of tubers using GC–MS. We detected 280 compounds, 113 of which were present at a different concentration in the tubers of the two genotypes. Hetero-grafting altered the level of 75 metabolites in CE3027 and 125 metabolites in CE3130 rootstock tubers. Furthermore, 29 compounds with scion-dependent concentrations were identified, which we assume are synthesised in leaves and transported to tubers.

## Materials and methods

### Plant materials and growth conditions

The diploid potato cultivars CE3027 and CE3130 were generously provided by Prof. R.G.F. Visser (Wageningen University and Research, Wageningen, The Netherlands) and propagated in vitro under standard conditions. Four-week-old plantlets were transferred to small pots and grown further in a greenhouse. Plant growth conditions are described in detail by Odgerel and Bánfalvi (2021).

### Grafting and morphological studies

The plants were grafted 2 weeks after planting the in vitro plantlets into pots. The stems of the plants served as scions and rootstocks were cut at approximately 2 cm above the soil with a diagonal cut. The scions were placed on the rootstocks and wrapped with rubber clips. The grafted plants were incubated under high humidity in a glassy box in a shaded area of the greenhouse. After 1 week, the grafted plants were transferred into larger pots (14 cm diameter top and 14 cm deep) and grown under ambient light (12–15 h/12–9 h dark) at 20–26 °C with regular watering. Two consecutive grafting experiments were carried out. In the first experiment, tuber formation was tested by carefully tipping the plants out of the pots together with the soil and re-planting them after counting the tubers. This experiment was carried out with 5–5 non-grafted control, 5–5 homo-grafted and 15–15 hetero-grafted plants. In the second experiment, 20–20 control non-grafted, 30–30 homo-grafted and 30–30 hetero-grafted plants were used. In both experiments, the tubers were harvested at the end of the vegetation period of the scions and visually evaluated for shape and colour.

### Metabolite extraction and profiling using GC–MS

#### Extraction

Freshly harvested tubers were washed and blotted dry with tissue paper. As described by Shepherd et al. (2007), the two opposite eighths were removed from each tuber leaving an approximately 1-cm thick radial slice in the middle. The tuber slices were ground in liquid nitrogen and stored at − 70 °C. 100 mg of the stored samples was used for metabolite analysis. Extraction was performed in 700 µl of GC-grade methanol. Ribitol (30 µl, 0.2 mg ml^−1^) and methyl nonadecanoate (60 µl, 0.4 mg ml^−1^) were added as internal standards. Samples were vortexed and incubated at 70 °C with continuous shaking at 1000 rpm for 15 min. MilliQ water (730 µl) and GC-grade chloroform (375 µl) were added. After vortexing, the samples were centrifuged at 13,000 rpm for 15 min. Aliquots of 150 µl of the supernatant (water phase) and 150 µl of the chloroform phase were taken for polar and non-polar metabolite analyses, respectively. Polar fractions were stored directly at − 70 °C until use, while the non-polar fractions were first vacuum dried and then re-dissolved in isohexane containing 50 ppm 2,6-di-t-butyl-4-methyl-phenol (BHT).

#### Water phase derivatisation

As described by Shepherd et al. (2007), the samples were vacuum-dried in 1.5-ml Eppendorf tubes and used for the derivatisation of the polar compounds with 40 µl of MEOX (methoxyamine hydrochloride) and 60 µl of MSTFA (N-methyl-N-(trimethylsilyl)trifluoroacetamide). The samples were incubated at 37 °C for 90 min with MEOX and at 37 °C for 90 min with MSTFA with continuous shaking at 300 rpm.

#### Chloroform phase derivatisation

Derivatisation of metabolites found in the chloroform phase was performed as described by Shepherd et al. (2007). Samples stored in BHT in 2-ml Eppendorf tubes were vacuum dried. As a post-extraction treatment, 400 µl of 1% (v/v) sulphuric acid in methanol, 1 ml of 5% (w/v) NaCl and 600 µl of chloroform were used; the solution was gently agitated and allowed to stand at room temperature for 15 min. The supernatant was discarded, and 600 µl of 2% (w/v) KHCO_3_ was added, the solution was gently agitated and then allowed to stand for 15 min. The supernatant was discarded, and the chloroform fraction vacuum dried. For derivatisation, 36 µl of chloroform, 7 µl of pyridine and 57 µl of MSTFA were added and the solution was incubated for 30 min at 37 °C with continuous shaking at 300 rpm.

#### Chromatography

The samples were analysed in a quadrupole type GC–MS system (Finnigan Trace GC/DSQ, Thermo Electron Corp., Austin, TX, USA) equipped with a low polarity phase, 5% diphenyl/95% dimethyl polysiloxane capillary column (TG-5MS, 30 m × 0.25 mm × 0.25 µm, Thermo Scientific, Waltham, MA, USA). The chromatograph used was Trace GC Ultra 2, with an AI/AS 3000 (version 2) autosampler. Sample volumes of 1 µl were injected with a split ratio of 10 ml min^−1^ at an injection temperature of 180 °C.

#### Mass spectrometry

Spectrometer Trace DSQ Ultra quadrupole mass spectrometer, electron source: EI, carrier gas: helium. Scans were made in positive ion mode, with the full scan in the mass range between 50 and 650 m/z. Ion source temperature 250 °C, MS transfer line 300 °C, flow 1 ml min^−1^. The temperature programme was 90 °C initial temperature for 2 min, a ramp for 15 min up to 165 °C; a hold time of 15 min; ramp for 6 min up to 320 °C.

#### Data transformation

The data were processed using Xcalibur Data System Software 1.4.1 SP3 (Thermo Finnigan, San Jose, CA, USA). The quantitative analysis was carried out based on the relative peak area using the peaks of the internal standards ribitol (water phase) and methyl nonadecanoate (chloroform phase) as one unit.

#### Metabolite identification

Before sample measurement, alkane standards (C7–C40) dissolved in n-hexane were measured. Based on the retention times of the homologous alkanes, the relative retention index (RI) was calculated for each peak based on the linear interpolation of retention times. Metabolite identification was based on the NIST MS Search 2.0 software and the calculated RIs.

### Starch assay

The extraction and enzymatic breakdown of starch to glucose were based on the study of Chow and Landhäusser (2004). The amount of glucose generated by α-amylase and amyloglucosidase treatment was determined by GC–MS using the same protocol as described in Sect. 2.3.2.

### Data analysis

The online MetaboAnalyst 5.0 (www.metaboanalyst.ca) statistical software was used for all statistical analyses. Relative amounts of data of identified and unidentified metabolites were normalised to the median and log-transformed for each analysis including the PCA (principal component analysis), PLS-DA (partial least-squares discriminant analysis), VIP (variable importance in projection), dendrograms, heat maps, box plots showing the results of one-way ANOVA with a post hoc Tukey’s HSD test at adjusted *p*-value of 0.05 and Student’s *t*-test (*P* ≤ 0.05). Based on Chong and Jun (2005), 1.5 was selected as the VIP cut off value.

## Results

### Morphology and tuberisation of grafted plants

**Fig. 1:**
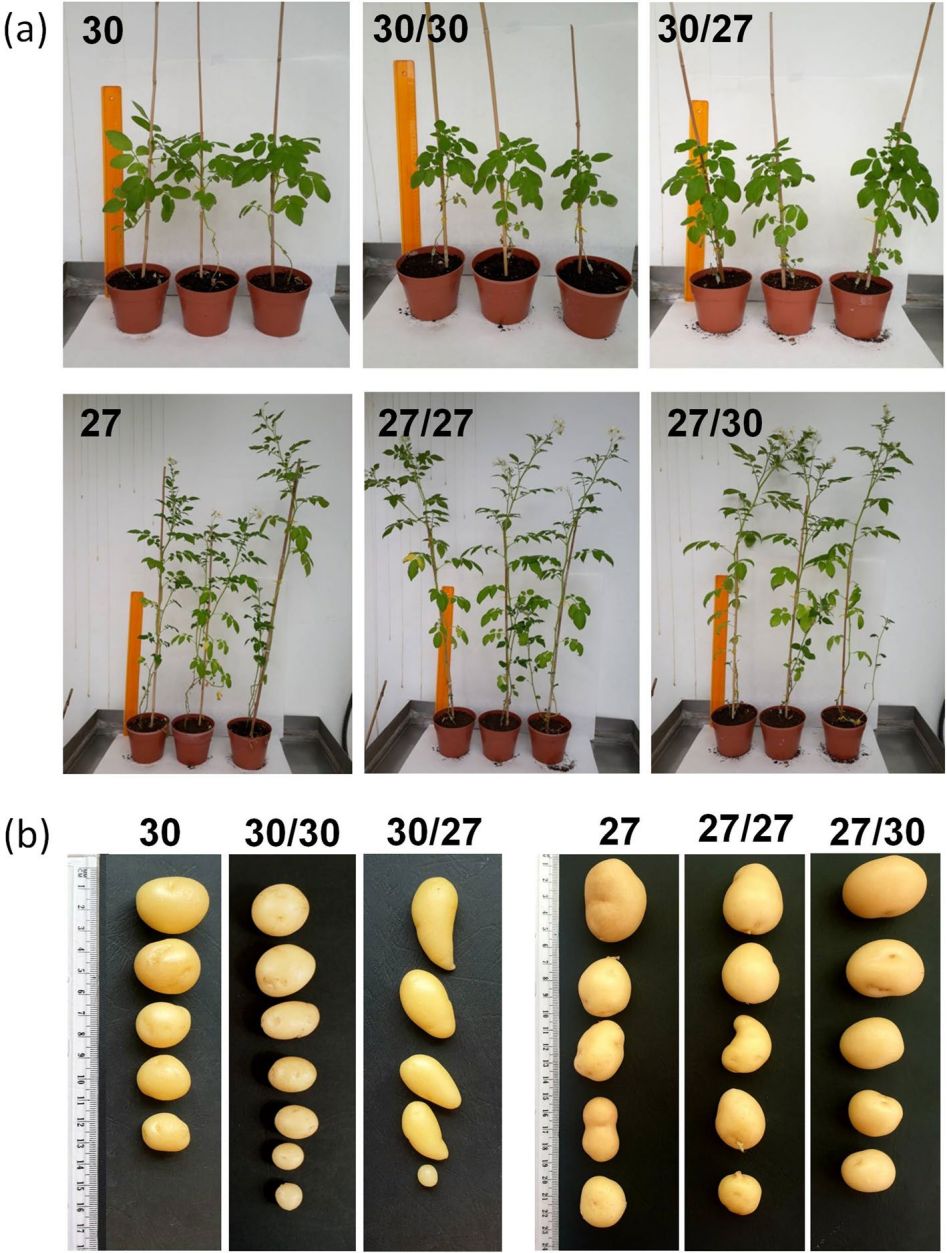
Canopy **a** and tuber morphology **b** of the early-maturing line CE3130 and the late-maturing line CE3027. Photos were taken on the plants 2 months after grafting. The size of the yellow ruler is 50 cm. Tubers were harvested at the end of the vegetation period of the scion. 30, non-grafted CE3130; 30/30, homo-grafted CE3130; 30/27; heterograft: CE3130 scion/CE3027 rootstock; 27, non-grafted control CE3027; 27/27, homo-grafted CE3027; 27/30, hetero-graft: CE3027 scion/CE3130 rootstock

CE3130 is a very early-maturing cultivar, while CE3027 is a very late-maturing potato line (Kloosterman et al., 2013). To test the morphology and tuberisation of the lines and the effect of hetero-grafting compared with non-grafted and homo-grafted controls, the plants were grown in a greenhouse and visually evaluated for height and flowering and shape and colour of tubers. Figure 1 shows that the CE3130 plants were much shorter than the CE3027 plants. Two months after grafting, all CE3027 plants produced flowers, while no flowers had yet developed on the CE3130 plants in our greenhouse conditions. The CE3130 tubers were all roundish, while several elongated forms were recognised among the CE3027 tubers. The grafting did not influence any of these differences between the two lines. The skin colour of the tubers was yellow in both lines.

Tuber formation was monitored starting in the sixth week after grafting. By this time, small tubers had developed on the CE3130 and homo-grafted CE3130/CE3130 plants. In contrast, even at 73 days after grafting, tubers had formed on only 80% of the CE3027 and CE3027/CE3027 homo-grafted plants. In both cases, the hetero-grafting altered the time of tuber initiation: the CE3130 scions accelerated, while the CE3027 scions delayed the tuberisation of the hetero-stock by approximately 3 weeks (Table 1).

**Table 1:** Tuberising plants in %

Grafts^a^	Days after planting			
	42	51	65	73
CE3027	0	0	0	80
CE3027/CE3027	0	0	20	80
CE3027/CE3130	0	0	53	100
CE3130	100	100	100	100
CE3130/CE3130	100	100	100	100
CE3130/CE3027	53	100	100	100

^a^Non-grafted and homo-grafted plants were tested in 5 parallel setups, while hetero-grafted plants were tested in 15 parallel setups

### Effect of grafting on tuber metabolome

**Fig. 2:**
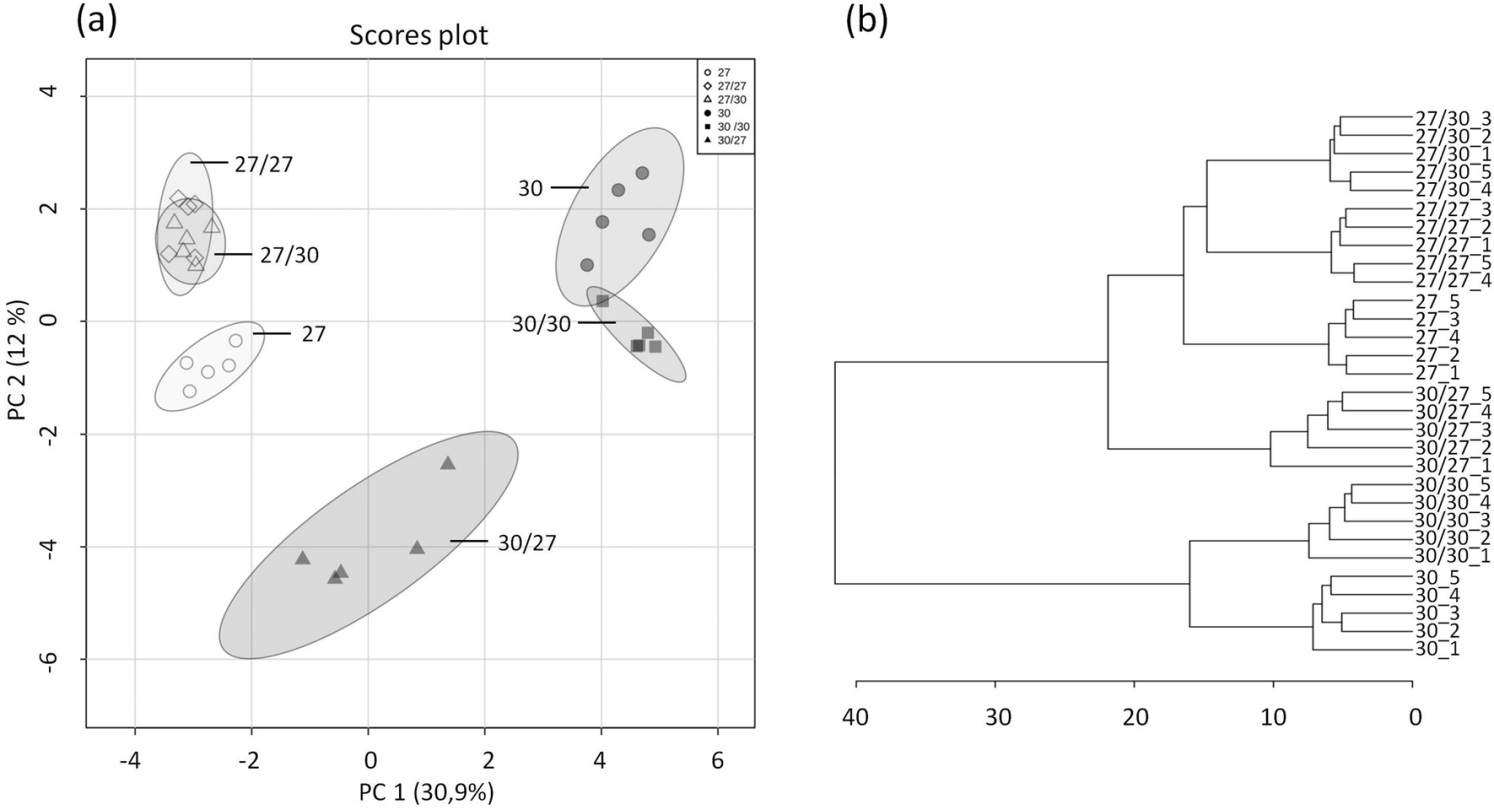
Differences in metabolite composition of tubers of CE3027 and CE3130 potato plants and their homo- and hetero-grafts. **a** PCA score plot, **b** dendrogram illustrating the hierarchical clustering of tuber types based on metabolite composition. Tubers were derived from the second experiment described in Materials and methods sub-Sect. 2.2. Data were obtained from 5 groups of tubers per type. Each group contained 5 slices with 2–3 cm in diameter cut from 5 tubers. The labels are as in Fig. 1

The metabolic composition of freshly harvested tubers was analysed using GC–MS in TIC (total ion chromatography) mode. Based on library data and authentic standards, 99 metabolites were identified and an additional 181 peaks were detected in the chromatograms. Principal component analysis (PCA) of the CE3027 and CE3130 tuber metabolite data revealed distinct profiles causing the two sample sets to cluster based on genotype. Homo-grafting did not cause a substantial change in the metabolite composition of CE3130/CE3130 tubers compared with the CE3130 control. In contrast, significant differences were detected in the tubers of CE3027/CE3027 homo-grafts and both hetero-grafts compared with the non-grafted controls. In particular, the effect of CE3027 on the metabolite content of the tubers formed on CE3130 rootstocks was evidenced by the PCA clustering of CE3027/CE3130 tubers with the CE3027/CE3027 tubers (Fig. 2a). Hierarchical clustering also highlighted the dominating influence of CE3027 scions on the metabolite content of tubers as it organised the plants into two main clades (CE3130 and its homo-graft and CE3027, its homo-graft and both hetero-grafts; Fig. 2b).

**Fig. 3:**
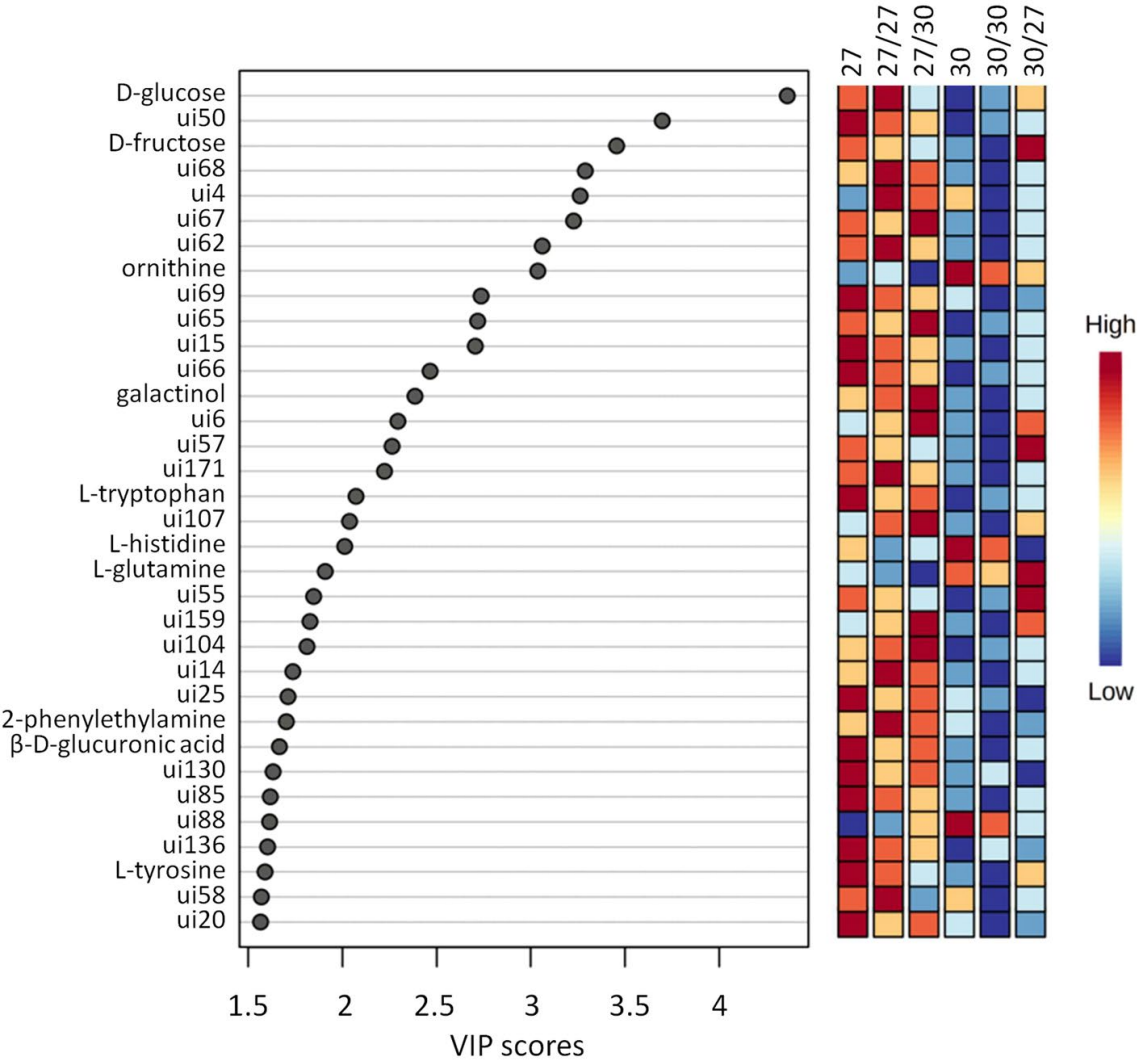
VIP score plot showing the top 34 (score > 1.5) most important metabolite features identified by PLS-DA. The statistical analysis was carried out with the same dataset as in Fig. 2. The labels are as in Fig. 1

To determine which compounds caused the major differences between the tubers of different plants, variable importance projection (VIP) plots by partial least squares-discriminant analysis (PLS-DA) were utilised. Thirty-four compounds had a VIP score higher than 1.5, however, only ten of them could be identified in this study: d-glucose, d-fructose, ornithine, galactinol, l-tryptophan, l-histidine, l-glutamine, l-phenylalanine, β-d-glucuronic acid and l-tyrosine (Fig. 3).

Concentrations of 113 metabolites (80 polar and 33 non-polar metabolites) were significantly (*P*** ≤ **0.05) different in CE3027 and CE3130 tubers. The homo-grafting influenced the amounts of 45 compounds in CE3027 and 33 compounds in CE3130 tubers, while the hetero-scions altered the level of 75 and 125 metabolites in CE3027 and CE3130 rootstock tubers, respectively (Supplementary Tables 1–5). CE3027 scions strongly affected the non-polar metabolite composition of tubers developed on CE3130 rootstocks as they altered the levels of 36 non-polar compounds, including 3-penten-2-one, linolenic acid, octadecane and 33 unidentified compounds (Supplementary Table 5).

**Fig. 4:**
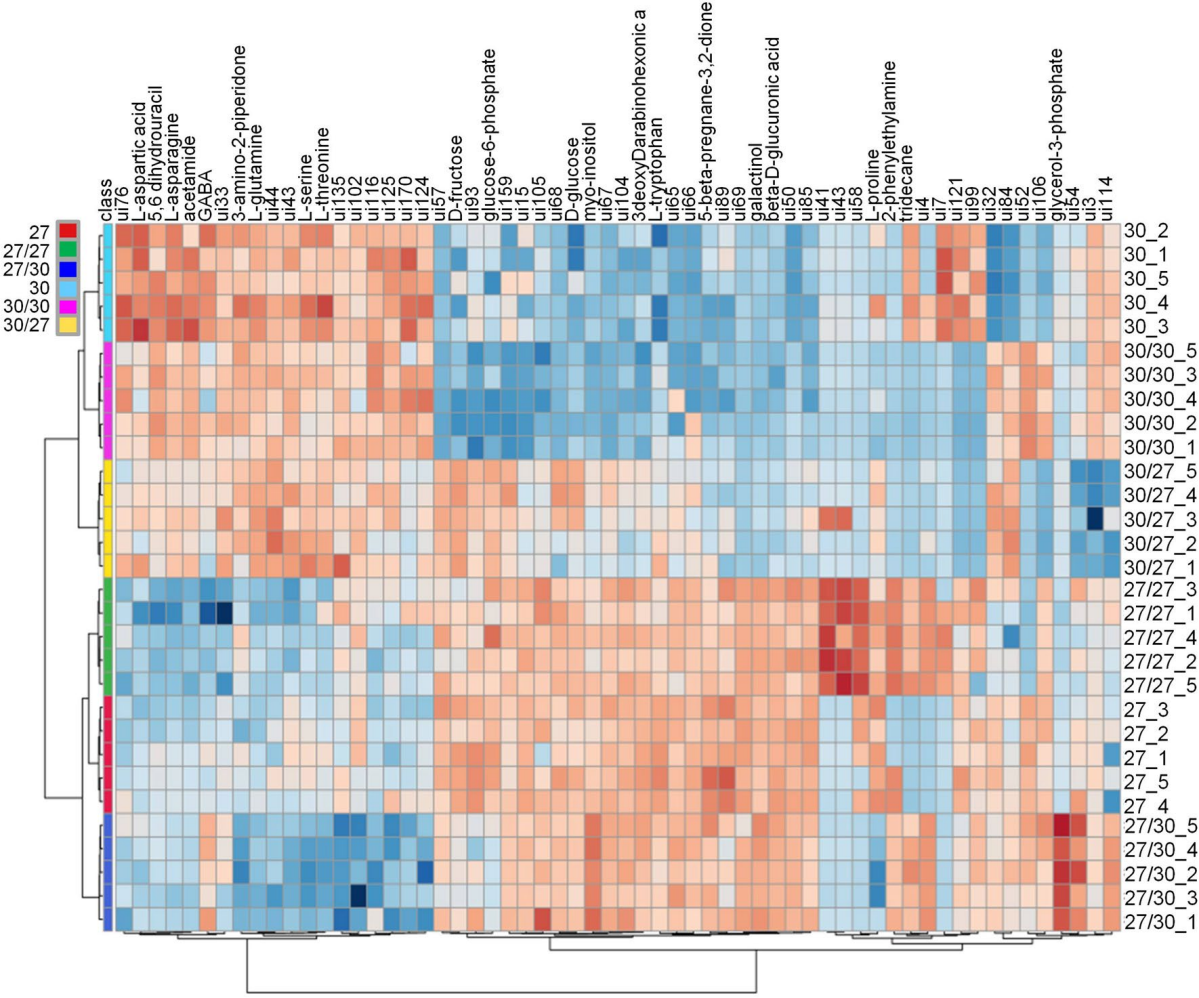
The heatmap of the top 60 metabolites differentiating CE3027, CE3027/CE3027, CE3027/CE3130, CE3130, CE3130/CE3130 and CE3130/CE3027 tubers. The statistical analysis was carried out with the same dataset as in Fig. 2. The labels are as in Fig. 1

Visualisation of the amounts of the compounds influenced by grafting in a heatmap partitioned the metabolites into main four groups: (1) influenced by both types of scions; (2) not influenced by CE3130 scion; (3) increased in CE3027 homo-grafts; (4) decreased in CE3130 homo-grafts. The heatmap of the top 60 metabolites differentiating CE3027 and CE3130 tubers and those developed on grafted plants is presented in Fig. 4.

Twenty-nine compounds had characteristic concentration differences between CE3027 and CE3130 and were influenced in amount by both genotypes as scions in the tubers of hetero-rootstocks compared with the tubers of the respective homo-graft (Table 2 and Supplementary Table 6).

**Table 2:** Metabolites with scion-dependent concentration

Metabolites	Fold changes	Fold changes
	CE3027/CE3130 CE3130/CE3130	CE3130/CE3027 CE3027/CE3027
Polar metabolites		
2-phenylethylamine	2.62	0.30
3-deoxy-d-arabino-hexonic acid	1.27	1.07
5.6-dihydrouracil	0.15	3.80
5β-pregnane-3.20-dione	1.69	1.33
acetamide	0.13	4.44
alloxanoic acid	0.18	2.20
β-alanine	0.22	2.14
β-d-glucuronic acid	2.73	0.37
d-fructose	10.25	9.92
d-sorbitol	0.73	1.32
galactinol	7.33	0.16
l-asparagine	0.18	4.45
l-aspartic acid	0.35	2.13
l-glutamine	0.06	11.43
l-serine	0.21	2.56
l-threonine	0.20	2.67
pipecolic acid	0.09	5.68
serotonin	0.28	2.71
ui4	2.63	0.01
ui15	2.90	0.34
ui34	0.12	5.27
ui37	0.79	1.96
ui40	0.31	2.61
ui44	0.13	6.44
ui50	8.22	0.10
ui53	0.26	2.86
ui54	1.04	1.54
ui65	3.07	1.96
ui66	1.29	1.57
ui69	7.68	0.31
ui76	0.20	2.60
ui85	2.04	1.85
ui99	3.53	0.23
ui104	3.16	0.48
ui105	1.72	1.28
Non-polar metabolites		
ui114	0.85	0.29
ui121	3.68	0.12

*ui* un-identified

### Concentration changes of selected metabolites

**Fig. 5:**
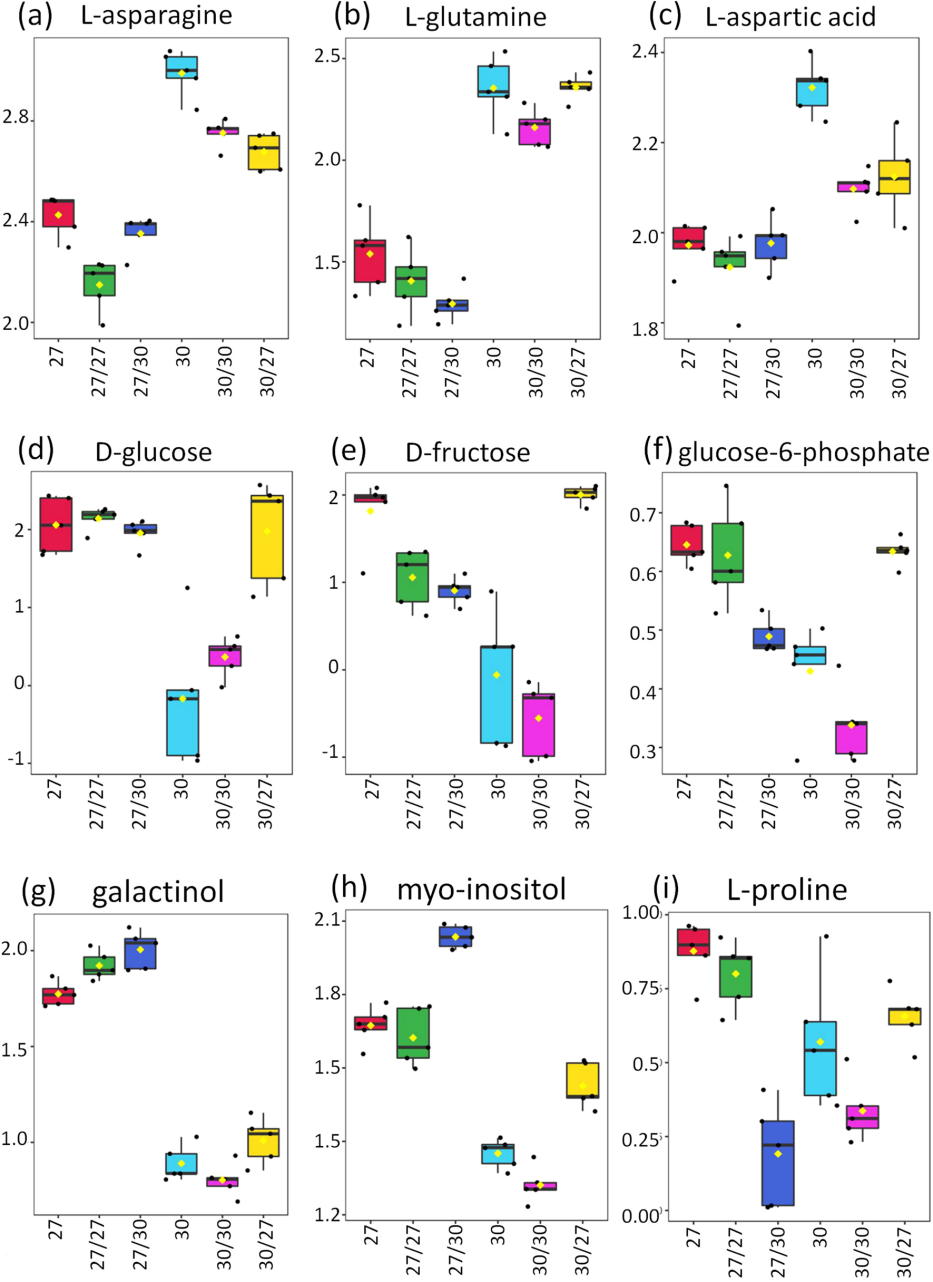
Relative abundances of nine metabolites present in different amounts in CE3027 and CE3130 tubers and in the tubers of grafted plants. The box plot includes the median (50% of all values above and 50% below), the first quartile (25% of all values below the quartile), the third quartile (25% of all values above the quartile), and whiskers indicating the highest and the lowest value of the data set. Outliers are dotted. The statistics is based on one-way ANOVA with a post hoc Tukey’s HSD test at adjusted p-value of 0.05. The labels are as in Fig. 1

Sucrose, citric acid, l-asparagine, l-glutamine and l-aspartic acid were present in the largest amounts in CE3027 and CE3130 tubers. Nevertheless, out of these compounds, only the concentrations of l-asparagine, l-glutamine and l-aspartic acid were different between the two types of tubers. These metabolites were present in lower amounts in CE3027 than in CE3130 tubers, reduced amounts in CE3027/CE3130 tubers and increased amounts in CE3130/CE3027 tubers compared with the homo- and non-grafted controls (Fig. 5a–c). d-glucose and d-fructose were the dominating monosaccharides in the tubers of both genotypes. However, there were significant differences in the concentrations of these two compounds in the tubers of CE3027 and CE3130, being higher in the tubers of the late-maturing CE3027 than in those of the early-maturing CE3130 genotype. Interestingly, while the CE3027 scions could increase the d-glucose and d-fructose content of the tubers formed on CE3130 rootstocks, the CE3130 scions could not influence the concentrations of these two compounds in CE3130/CE3027 tubers (Fig. 5d, e). A similar tendency was observed for glucose-6-phosphate (Fig. 5f). Galactinol is derived from myo-inositol and is a precursor of the raffinose family of oligosaccharides, which serve as desiccation protectants and storage sugars (Nishizawa et al., 2008). The galactinol concentration was higher in CE3027 than in CE3130 tubers and was increased in CE3027/CE3130 and decreased in CE3130/CE3027 tubers compared with their respective non-grafted and homo-grafted controls (Fig. 5g). The concentration of myo-inositol and the trends among the different tuber types were similar to that of galactinol. However, the influence of CE3130 on the CE3027 rootstock was less pronounced than in the case of galactinol (Fig. 5h). Proline plays a crucial role in maintaining the metabolism and growth of plants under abiotic stress conditions (reviewed by Ghosh et al., 2022). Therefore, we analysed the proline content of tubers and found that it was higher in CE3027 tubers than in CE3130 tubers and was not significantly influenced by grafting (Fig. 5i).

The starch content of potato tubers ranges from 10 to 25% (Schönhals et al., 2016). Despite this wide range, no significant (*P*** ≤ **0.05) differences in the starch content of the CE3027 and CE3030 tubers, nor the tubers of grafted plants, were detected. The average starch content of all six categories of tubers was around 22 ± 5% (Supplementary Fig. 1).

## Discussion

While grafting technology has become widely implemented, the effects of inter-cultivar grafting on the metabolite composition of potato tubers are still largely unknown. Odgerel and Bánfalvi (2021) analysed two commercial potato cultivars with relatively similar tuber initiation times, while in the current work, very early- and very late-maturing potato lines were grafted. The previous study was extended only to 31 polar metabolites. In this study, 78 polar and 21 non-polar metabolites were identified and an additional 181 unidentified compounds were included in the statistical analysis.

Interspecies grafting experiments, especially those with tomato/potato, nicely exemplified the morphological influence of rootstocks on scions. Kudo and Harada (2007) reported a graft-transmissible RNA from tomato rootstock changing the leaf morphology of potato scion. Grafting experiments with different tomato and potato cultivars, except for the flower initiation time, demonstrated many morphological changes (e.g., plant height, leaf number, branch number, fruit size, etc.) in tomato scions (Arefin et al., 2019). In contrast, Zhang et al. (2019) did not find significant morphological differences in tomato scions. Still, they did find some in the potato rootstock where the stolon number, stolon length and tuber number were decreased. Although the early- and late-maturing potato lines that we analysed were morphologically very different, no influence of hetero-grafting on the height of the plants, flowering time or tuber shape was detected. In contrast, the scion strongly influenced the time of tuber initiation, which supports the previous finding that tuber formation is triggered by leaf-derived mobile signals (Navarro et al., 2011). Plantenga et al. (2019) demonstrated that flower bud development is not improved by tuber sink removal in the CE3027 and CE3130 genotypes. In the present study, we found that as a rootstock, CE3130 cannot impede the early flowering of CE3027 and vice versa, CE3027 as a rootstock cannot induce flowering in CE3130 scions, indicating that there is no genetically determined signal arising from the rootstock, which could influence the scion’s flowering time.

Homo-grafted plants were applied as controls in the experiments. Although to a lesser extent than in the tubers of the hetero-grafts, differences in the concentration of certain compounds compared with the non-grafted controls were also detected in the tubers of homo-grafted plants. Since the tubers were harvested 3 and 4 months after grafting, it is doubtful that the effect of grafting, as a wounding, would cause these differences. It is much probable that the biological variation of tubers and the lower analytical reproducibility of specific compounds are reflected by these differences as described in the literature, for example, in the case of *Arabidopsis* (Fiehn et al., 2000).

Tuberisation is triggered by mobile signals transported from leaves to stolon tips (reviewed by Zierer, 2021). Using ^14^C-labelling techniques, Rachow-Brandt & Kollmann (as early as 1992) demonstrated that the rates of assimilate transport between scion and rootstock correlate with the number of sieve tubes, indicating that the route of translocation is via the phloem. Soluble carbohydrates, most notably sucrose, are potent inducers of tuberisation, and storage organs, in general, are net importers of assimilates (reviewed by Fernie & Willmitzer, 2001). Our experiments identified many compounds with similar levels in tubers of hetero-grafts as in tubers of scions (i.e., l-asparagine, l-glutamine, l-serine, l-threonine, galactinol, l-aspartic acid, β-d-glucuronic acid, etc.). We assume that these compounds are transported from the source leaves to the tubers.

Although the tubers of early- and late-maturing genotypes did not differ in starch content, the fructose, glucose and glucose-6-phosphate concentrations in the tubers of the late-maturing CE3027 were higher than in those of the early-maturing CE3130 genotype. The CE3027 scion could increase these compounds’ concentrations in CE3130 rootstock tubers, while the CE3130 scion could not alter the concentrations of these compounds in CE3027 tubers. In an enzymatic step, catalysed by invertases, glucose and fructose are derived from sucrose. The hexoses then can be phosphorylated and directed to starch synthesis. The protein composition of tubers, however, continuously changes during their development, and by the end of the vegetation period, only a few highly abundant proteins, such as, for example, enzymes involved in starch synthesis or the storage protein, patatin, remain in substantial amounts (reviewed by Fernie & Willmitzer, 2001). We harvested the tubers at the end of the vegetation period when their development was already complete, but the starch synthesis still might be active. Thus, the sucrose, which was transported from leaves probably in a higher amount from the higher plant CE3027 than from the smaller plant CE3130, could be cut by invertases into glucose and fructose and the glucose phosphorylated to yield glucose-6-phosphate at higher amounts in the tubers of CE3130 rootstocks of hetero- than homo-grafted plants.

An unexpected observation was the altered concentrations of many non-polar metabolites in the CE3027/CE3130 tubers compared with the CE3130/CE3130 tubers. The total lipids represented approximately 0.1–0.5% of potato tuber fresh weight, and these were mainly composed of linoleic- and linolenic acid (70–75%). Major lipids are associated with tuber membranes (Ramadan & Oraby, 2016); however, other non-polar metabolites such as, for example, fucosterol, sitosterol, octacosanoic acid, etc., have also been detected in potato tubers (Shepherd et al., 2015). Lipids are transported and play a wide diversity of roles in the plant life cycle (reviewed by Wang & Chapman, 2013). Since the number of non-polar compounds altered in concentration was much fewer in CE3130/CE3027 than in CE3027/CE3130 tubers (8 versus 36), a similar explanation described for fructose, glucose and glucose-6-phosphate, namely that more metabolites are transported to tubers from larger than smaller plants, may be valid also for non-polar compounds. Nevertheless, the identification of non-polar metabolites is needed to understand better the mechanisms underlying the concentration differences in the tubers of homo- and hetero-grafted plants.

## Conclusion

We found that the metabolite composition of tubers of the late maturing potato genotype CE3027 and that of the early maturing potato genotype CE3130 is largely different. Grafting experiments showed that the concentration of majority of the detected 280 compounds can be influenced by hetero-scions. Twenty-nine compounds with similar levels in tubers of hetero-grafts as in tubers of scions were detected suggesting that these compounds are synthesised in leaves and transported to tubers. Concentrations of several un-identified non-polar metabolites in the CE3027/CE3130 tubers were altered compared with the control tubers. Identification of these compounds may result in better understanding the processes associated with quality traits of potato tuber.

## Supplementary Information

Below is the link to the electronic supplementary material.

Supplementary file1 (XLSX 31 KB)

Supplementary file2 (PDF 116 KB)

## Data Availability

The datasets generated during and/or analyzed during the current study are available at Metabolights (https://www.ebi.ac.uk/metabolights/) under the study identifier MTBL4445.
