# Supplementary material for: Metabolite profiling of tubers of an early- and a late-maturing potato line and their grafts
Source: Metabolomics. 2022 Nov 5;18(11):88. doi: 10.1007/s11306-022-01950-3 (PMC9637070; doi:10.1007/s11306-022-01950-3)
Supplement: Supplementary file 2 — Supplementary file2 (PDF 116 KB) [file 11306_2022_1950_MOESM2_ESM.pdf]

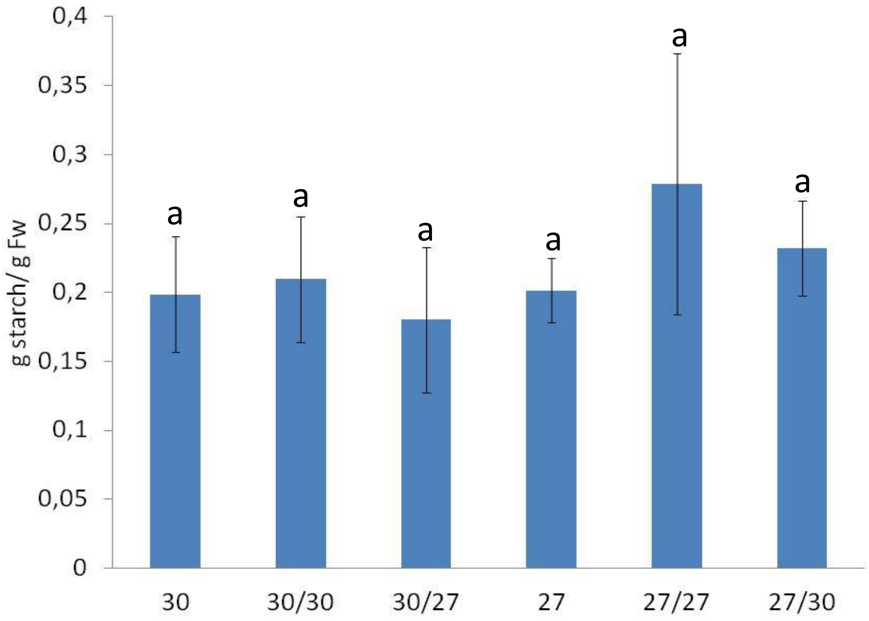

Fig. S1 Starch content of tubers. The same tuber powder was used for starch content analysis as for metabolic profiling. Statistical analysis was performed with one-way ANOVA with a *post hoc* Tukey HSD test. However, no significant difference between any of the samples was detected. Fw. fresh weight; 30, non-grafted CE3130; 30/30, homo-grafted CE3130; 30/27; heterograft: CE3130 scion/CE3027 rootstock; 27, non-grafted control CE3027; 27/27, homo-grafted CE3027; 27/30, hetero-graft: CE3027 scion/CE3130 rootstock
